# Elevated platelet count is a negative predictive and prognostic marker in locally advanced rectal cancer undergoing neoadjuvant chemoradiation: a retrospective multi-institutional study on 965 patients

**DOI:** 10.1186/s12885-018-5022-1

**Published:** 2018-11-12

**Authors:** Claudio Belluco, Marco Forlin, Paolo Delrio, Daniela Rega, Maurizio Degiuli, Silvia Sofia, Matteo Olivieri, Salvatore Pucciarelli, Matteo Zuin, Giovanni De Manzoni, Alberto Di Leo, Stefano Scabini, Luigi Zorcolo, Angelo Restivo

**Affiliations:** 1 Department of Surgical Oncology, CRO-IRCCS, National Cancer Institute, Aviano Via Franco Gallini 2, 33081 Aviano, Italy; 2 Colorectal Surgical Oncology, National Cancer Institute – IRCCS – G. Pascale Foundation, Naples, Italy; 3 School of Medicine, Department of Oncology, Head, Digestive, University of Torino, Torino, Italy; 4 Surgical Oncology, San Luigi University Hospital, Orbassano, Torino, Italy; 5 Department of Surgical, Oncological and Gastroenterological Sciences, University of Padova, Padova, Italy; 6 Department of Surgery, General and Upper G.I., Surgery Division, University of Verona, Verona, Italy; 7 Oncologic Surgery and Implantable Systems Unit, Department of Emergency, IRCCS San Martino IST, Genoa, Italy; 8 Colorectal Surgery Unit, Department of Surgical Sciences, University of Cagliari, Cagliari, Italy

**Keywords:** Platelets, Thrombocytosis, Rectal Cancer, Neoadjuvant chemoradiation, Predictive factors, Prognostic factors, Pathological response, Aspirin

## Abstract

**Background:**

In patients with locally advanced rectal cancer treated by neoadjuvant chemoradiation, pathological complete response in the surgical specimen is associated with favourable long-term oncologic outcome. Based on this observation, nonoperative management is being explored in the subset of patients with clinical complete response. Whereas, patients with poor response have a high risk of local and distant recurrence, and appear to receive no benefit from standard neoadjuvant chemoradiation. Therefore, in order to develop alternative treatment strategies for non responding patients, predictive and prognostic factors are highly needed. Accumulating clinical observations indicate that elevated platelet count is associated with poor outcome in different type of tumors. In this study we investigated the predictive and prognostic impact of elevated platelet count on pathological response and long-term oncologic outcome in patients with locally advanced rectal cancer undergoing neoadjuvant chemoradiation.

**Methods:**

A total of 965 patients were selected from prospectively maintained databases of seven Centers within the SICO Colorectal Cancer Network. Patients were divided into two groups based on a pre-neoadjuvant chemoradiation platelet count cut-off value of 300 × 10^9^/L identified by receiver operating characteristic curve considering complete pathological response as the outcome.

**Results:**

Complete pathological response rate was lower in patients with elevated platelet count (12.8% vs. 22.1%, *p* = 0.001). Mean follow-up was 50.1 months. Comparing patients with elevated platelet count with patients with not elevated platelet count, 5-year overall survival was 69.5% vs.76.5% (*p* = 0.016), and 5-year disease free survival was 63.0% vs. 68.9% (*p* = 0.019). Local recurrence rate was higher in patients with elevated platelet count (11.1% vs. 5.3%, *p* = 0.001), as higher was the occurrence of distant metastasis (23.9% vs. 16.4%, *p* = 0.007). At multivariate analysis of potential prognostic factors EPC was independently associated with worse overall survival (HR 1.40, 95% CI 1.06–1.86), and disease free survival (HR 1.37, 95% CI 1.07–1.76).

**Conclusions:**

In locally advanced rectal cancer elevated platelet count before neoadjuvant chemoradiation is a negative predictive and prognostic factor which might help to identify subsets of patients with more aggressive tumors to be proposed for alternative therapeutic strategies.

## Background

In locally advanced (T3–4 or N+) mid-distal rectal cancer (LARC), neoadjuvant chemoradiation therapy (CRT) before radical surgery including total mesorectal excision (TME) reduces the risk of local recurrence, and is considered standard treatment [1–3].

However, patients undergoing this multimodality treatment are exposed to the risk of perioperative morbidity and mortality, long-term bowel, bladder, and sexual dysfunction, and permanent colostomy [4, 5].

Pathological complete response (pCR) in the surgical specimen is obtained in up to one-third of LARC patients treated by neoadjuvant CRT, and is associated with favourable long-term oncologic outcome [6, 7]. Based on these observations, nonoperative management is being explored in the subset of patients with clinical complete response after CRT [8–11].

On the other hand, LARC patients with poor response to CRT have a high risk of local and distant recurrence, and appear to receive no benefit from standard neoadjuvant CRT.

Therefore, in order to develop alternative treatment strategies both for responding and not responding patients, predictive and prognostic factors are highly needed.

Extensive experimental evidence shows that platelets (PLT) have a crucial role in tumor progression and metastasis through diverse mechanisms, including promotion of epitelial-to-mesenchymal transition, protection of cancer cells from immune surveillance, negotiation of cancer-cell arrest in the micro-vasculature, and stimulation of angiogenesis [12–15]. Moreover, a feed-forward loop wherein tumor and host tissue thrombopoietic cytokines lead to PLT count increasing, which in turn promotes tumor growth, has been demonstrated [16].

Elevated platelet count (EPC) is frequently observed in subsets of patients with cancer, and accumulating clinical observations indicate that thrombocytosis associates with poor outcome in different type of tumors, including colorectal cancer [17–23]. However, at present few studies have examined the predictive and prognostic significance of EPC in rectal cancer undergoing neoadjuvant CRT.

## Methods

### Study design and objectives

This was a retrospective cohort study aimed to investigate the impact of EPC before neoadjuvant CRT on pCR rate, and long-term oncologic outcome in a large series of LARC patients, consecutively treated in high-volume Referral Centers for Colorectal Surgery, between January 2000 and December 2016. The study was approved by the Institutional Review Board of all participating Centers (coordinating Center ethics committee’s reference number CRO-2015-13). All items required by STROBE checklist for reports of observational studies have been included. Clinical and pathological information were retrieved from prospectively maintained electronic databases of 7 Italian Centers from the SICO - Colorectal Cancer Network collaborative study group. The clinical records of selected patients were merged and reviewed.

### Study population

Patients were included in the study if the following criteria were met: histological proved adenocarcinoma of the rectum located up to 12 cm from the anal verge (AV), pretreatment clinical stage II or III (cT3–4 and or cN+), no history of previous cancer, preoperative long course CRT.

The following clinical pretreatment data were considered for both groups: gender; age; distance of the tumor from the anal verge; cTNM stage; time interval between completion of CRT and surgery.

Initial clinical local stage was assessed by pelvic MRI or endorectal ultrasound, alternatively or in combination. Pretreatment staging always included physical examination, colonoscopy, abdominal and chest CT scan.

### Neoadjuvant treatment

Neoadjuvant treatment included external beam radiotherapy delivered with a total dose of at least 45 Gy administered over 5 weeks (25 fractions of 1.8 Gy/daily) and in most cases with a concomitant boost of 5,4 Gy for a total dose of 50,4 Gy. Concomitant chemotherapy was based on 5-FU either in a daily oral preparation (Capecitabine 1650 mg/m2/d) taken during the radiation period, in bolus infusion (5-FU 325 mg/m2/d × 5 days) during weeks 1 and 5, or as continuous infusion for 5 days per week over the entire 5 week radiation period (5-FU 250 mg/m2/d).

### Outcome measures

In order to overcome the limitation of the retrospective nature of the study, we selected pCR as primary endpoint since it is a strong independent prognostic factor of oncologic outcomes and is not affected by confounding factors depending on the subsequent history of the patients, such as for example, adjuvant treatment and time and quality of surgery for metacronous metastasysis.

pCR was defined as absence of any tumour cells at microscopic examination of the resected specimen on final pathology after surgery. Any tumor downstaged to pT0-T1 N0 was defined as good pathological response. All the other histopathological conditions, including partial downstaging were defined as incomplete pathological response.

Overall survival (OS) was calculated as the time from surgical resection to death from any cause, and disease free survival (DFS) was defined as time from surgical resection to tumor recurrence.

### Statistical analysis

Based on pre-neoadjuvant CRT blood samples data, patients were divided in two groups according to a PLT count cut off of 300 × 10^9^/L. This value was chosen by drawing a receiver operating characteristic (ROC) curve, considering the achievement of pCR as the outcome, and calculating the maximum level of the relative Youden Index. This corresponded to a PLT count value of 300 × 10^9^/L (Sensitivity 54%, Specificity 66%).

Difference between the groups were analysed by Fisher exact test for categorical variables, while continuous variables were tested by two independent sample T tests. Continuous values are expressed in mean and standard deviation.

A multivariate analysis including all available pretreatment data was also performed by binary logistic regression with pCR as dependent variable. Distance from anal verge and Interval before surgery were transformed into two categorical values before multivariate analysis execution:

- Distance from anal verge < 5 cm or > 5 cm, because precedent studies had already shown its correlation with pCR; [24]
- Interval before surgery < 8 weeks or > 8 weeks, because this cut-off have already shown its correlation with pCR and is currently used in clinical practice as the preferred waiting time lower limit [25].

Kaplan-Meier estimates and log-rank tests were used to assess the association of EPC with OS and DFS. A multivariate analysis for survival was performed by Cox proportional hazards regression, adjusting for sex (male vs. female), age, preoperative primary tumor (cT 1–2 vs. cT3–4) and lymph node (cN0 vs. cN+) stage, type of surgery (anterior resection/Hartmann vs. abdominoperineal resection/proctocolectomy vs. full thickness local excision), pre-CRT platelets count (< 300 × 10^9^/L vs. > 300 × 10^9^/L), interval to surgery (< 8 weeks vs. > 8 weeks), and distance from anal verge (< 5 cm vs. > 5 cm). To adjust for possible differences within participating centers, this variable was initially included in the multivariate model as a possible confounding variable, and no significant differences were observed. Proportionality of hazards assumption was satisfied by the Schoenfeld residuals method. A *p* value < 0.05 was considered statistically significant.

Statistical analysis was conducted using Stata 13.0 software (*Stata Statistical Software: Release 13*. College Station, TX: Stata Corp LP).

## Results

### Patients demographics and EPC distribution

A total of 965 patients (617 men, 348 women; median age 65 yrs) were selected for the study. EPC (PLT count > 300 × 10^9^/L) before neoadjuvant CRT was observed in 296 (30.7%) patients. No significant differences based on EPC status were observed for mean age and variables known to be correlated with pCR, namely distance of the tumour from the anal verge, preoperative stage T and N, and interval time before surgery. Of notice, EPC was significantly more frequent in female patients (Table 1).

**Table 1:** Clinico-pathological and treatment characteristics according to platelets count before neoadjuvant chemoradiation in 965 patients with locally advanced rectal cancer

	Platelet count				
Variable	< 300 × 10^9^/L		≥ 300 × 10^9^/L		*p*
Gender					
Women	220	32.88%	128	43.24%	0.002
Men	449	67.12%	168	56.76%	
Age (mean, 95% CI)	64.27 yrs	63.46–65.08	63.18 yrs	61.87–64.40	0.155
Distance from anal verge (mean, 95% CI)	6.24 cms	6.04–6.44	5.99 cms	5.69–6.29	0.169
Interval to surgery (mean, 95% CI)	8.83 weeks	8.50–9.16	8.75 weeks	7.99–9.51	0.829
Clinical primary tumor stage					
cT1	4	0.60%	3	1.01%	0.513
cT2	48	7.17%	22	7.43%	
cT3	562	84.01%	239	80.74%	
cT4	55	8.22%	32	10.81%	
Clinical lymph node stage					
cN0	254	37.97%	118	39.86%	0.576
cN+	415	62.03%	178	60.14%	
Type of surgery					
LAR	515	76.98%	222	75%	0.790
APR	114	17.04%	58	19.59%	
LE	29	4.33%	11	3.72%	
Other procedures	11	1.64%	5	1.69%	
Pathological primary tumor stage (ypT)					
ypT0	155	22.12%	41	12.84%	< 0.001
ypTis	6	0.90%	1	0.34%	
ypT1	74	11.06%	19	6.42%	
ypT2	168	25.11%	91	30.74%	
ypT3	257	38.42%	130	43.92%	
ypT4	9	1.35%	14	4.73%	
Pathological lymph node stage (ypN)					
ypN0	516	77.13%	211	71.28%	0.052
ypN+	153	22.87%	85	28.72%	

*LAR* Low anterior resection, *APR* Abdominoperineal resection, *LE* full thickness local excision

### PLT count and pathological response to neoadjuvant CRT

The main outcome of interest, rate of pCR, resulted significantly lower in patients with EPC (12.84% vs. 22.12%, *p* < 0.001). This difference was even more evident when considering “good pathological response” as the outcome, 17.43% in EPC patients compared to 32.99% in no-EPC patients (*p* < 0.001).

The independent correlation between platelet count and pCR was confirmed by multivariate analysis including other known prognostic factors for pCR (Table 2).

**Table 2:** Multivariate analysis (Binary Logistic Regression) using complete pathological response (pCR) to neoadjuvant chemoradiation as dependent variable in 965 patients with locally advanced rectal cancer

	Reference	Odds Ratio	(CI 95%)	*p*
Age		0.99	0.98–1.01	0.497
Sex	Female	1.11	0.80–1.56	0.533
cT	I-II	0.58	0.34–0.99	0.045
cN	0	1.05	0.75–1.46	0.797
Platelets count	< 300 × 10^9^/L	0.51	0.34–0.75	0.001
Distance from anal verge	< 5 cms	1.48	1.06–2.08	0.022
Interval between CRT and Surgery	< 8 weeks	1.35	0.93–1.97	0.115

*cT* Clinical primary tumor stage, *cN* Clinical lymph node stage, *CRT* chemoradiation

### Long-term oncologic outcome according to pathological response and PLT count

Mean follow-up for the entire patient population was 50.1 (± 1.1) months and it was comparable between EPC and no-EPC patients (51.6 ± 2.0 months, and 49.5 ± 1.3 months).

According to pathological response, 5-year OS was 86.1% for pCR patients compared to 71.5% for no-pCR patients (*p* = 0.002), and 5-year DFS was 81.9 and 63.8%, respectively (*p* < 0.001).

Local recurrence rate was significantly higher in EPC patients (11.15% vs. 5.38%, *p* = 0.001), as higher was the chance of distant relapse (23.9% vs. 16.4%, *p* = 0.007).

**Fig. 1:**
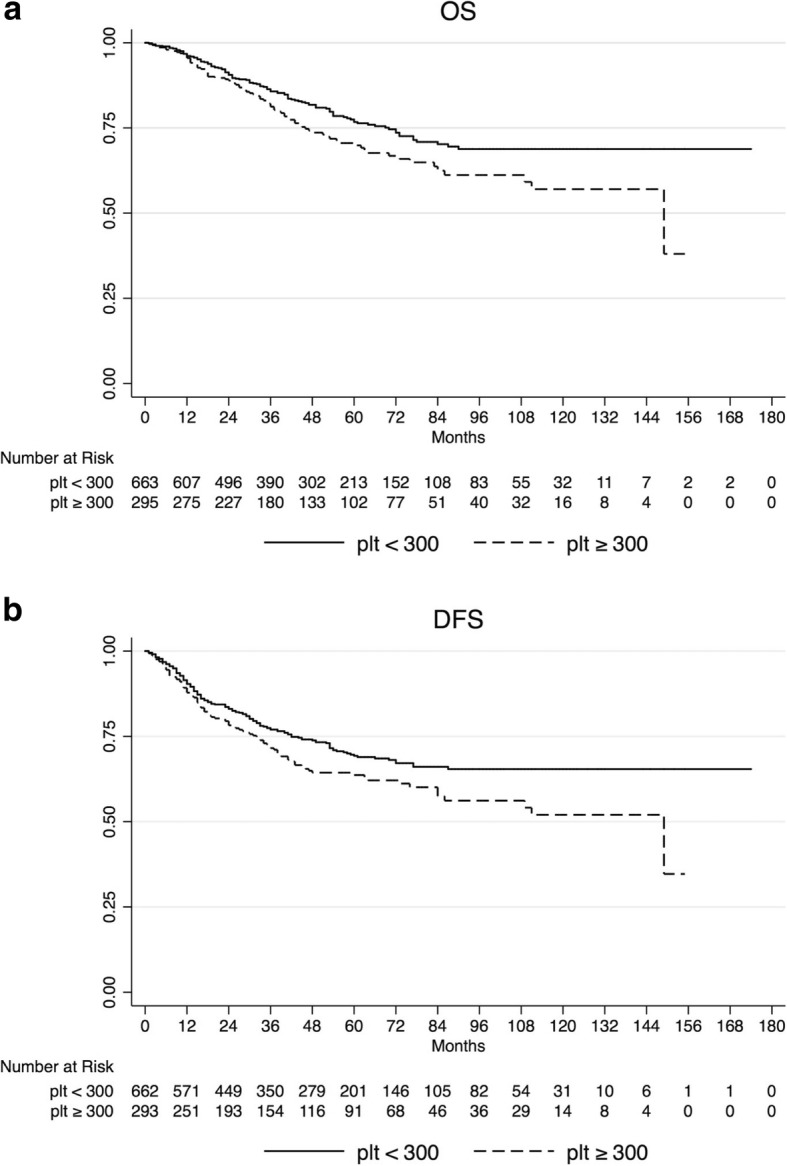
Kaplan-Meier estimates for overall survival (OS) (**a**), and disease-free survival (DFS) (**b**) according to platelet count before neoadjuvant chemoradiation in 965 patients with locally advanced rectal cancer

This translated also in a significantly worse survival outcome for these patients. Five-year OS was 69.5% for EPC patients compared to 76.5% for no-EPC patients (*p* = 0.016), and 5-year DFS was 63.0% and 68,9%, respectively (*p* = 0.019) (Fig. 1).

At multivariate analysis, after adjusting for other potential prognostic factors EPC was independently associated with worse OS (HR 1.40, 95% CI 1.06–1.86), and DFS (HR 1.37, 95%CI 1.07–1.76). (Table 3).

**Table 3:** Multivariate analysis of prognostic factors in 965 patients with locally advanced rectal cancer undergoing neoadjuvant chemoradiation

	Overall Survival				Disease-Free Survival		
	Reference	HR	(CI 95%)	*p*	HR	(CI 95%)	*p*
Age		1.04	1.03–1.06	0.000	1.03	(1.01–1.04)	0.000
Sex	Female	0.79	0.59–1.06	0.113	0.82	(0.63–1.06)	0.127
cT	I-II	1.58	0.88–2.84	0.127	1.18	(0.74–1.88)	0.495
cN	0	0.93	0.71–1.23	0.634	1.07	(0.84–1.37)	0.57
Platelets count	< 300 × 10^9^/L	1.45	1.10–1.92	0.009	1.39	(1.09–1.79)	0.009
Distance from anal verge	< 5 cms	0.62	0.47–0.81	0.001	0.59	(0.46–0.75)	0.000
Interval between CRT and Surgery	< 8 weeks	1.04	0.78–1.38	0.798	1.18	(0.91–1.54)	0.213

*HR* hazard ratio, *cT* Clinical primary tumor stage, *cN* Clinical lymph node stage, *CRT* chemoradiation

## Discussion

In the present study, we investigated the significance of platelet counts before neoadjuvant CRT in 965 LARC patients. To the best of our knowledge, this is the largest series published in the literature on this specific matter. Our findings indicate that EPC before treatment is a negative predictive and prognostic factor in patients with rectal cancer submitted to CRT.

The prevalence of EPC reported in studies on colorectal cancer patients varies between 8.0 and 49.8% depending on the defined cut-off. We decided to propose our own cut off value as it is still difficult to define a single best cut-off value for platelet count to be considered normal and or safe.

As reported in a recent meta-analysis, including studies investigating the prognostic significance of pretreatment platelet count in patients with colorectal cancer, the considered cut-off value varies from as low as 267 × 10^9^/L to as high as 450 × 10^9^/L, with the value of 300 × 10^9^/L being the minimum limit to maintain a statistically significance [26].

In this regard our study, being the largest published until now on this topic, actually serves to confirm an important prognostic significance of platelet count as well as to propose a shared cut-off value to use in clinical practice for identification of a subgroup of at risk patients.

In our data the prevalence of EPC (defined in this study as platelet count > 300 × 10^9^/L) was 30.7%. Interestingly, in our cohort of patients EPC was significantly more frequent in female patients. Similar findings have been reported by others [27, 28], and could be explained by the notion of baseline platelet counts and reactivity being higher in women compared to men [29–31]. However, the molecular mechanism of this biological phenomenon is not known.

Multivariate analysis showed that low platelet count before neoadjuvant CRT was an independent positive predictive factor for pCR, with an odd ratio of 1.92 (CI 95% 1.30–2.83). Our results are consistent with the data reported by others. Kim et al. in a series of 314 patients with locally advanced rectal cancer found that pCR was achieved in 3.0% of patients with pre-CRT platelets count > 370 × 10^9^/L compared to 14.4% of patients with platelets count < 370 × 10^9^/L (*p* = 0.01). Moreover, at multivariate analysis EPC was an independent negative predictive factor for pCR with an odd ratio of 5.48 [32]. Lee et al. recently reported in 291 consecutive LARC patients that, using a PLT count cut off value of 370 × 10^9^/L measured before CRT, pCR was achieved in 4.8% of the 41 cases with EPC compared to 20.8% of the 250 cases with not EPC (*P* < 0.05) [33]. In addition, Steele et al. in a small study set of 51 patients with stage II and III rectal adenocarcinoma receiving neoadjuvant CRT, found that patients with PLT counts < 300 × 10^9^/L were significantly more likely to exhibit a good or complete pathological response. (42.3% vs. 12,0%; *P* = 0.015) [34].

The results of our univariate and multivariate survival analysis supports the evidence that EPC associates with poor oncologic outcome in LARC patients undergoing neoadjuvant CRT. In our series, comparing patients with EPC with not EPC the 5-year OS was 69.5% vs. 76.5% (*p* = 0.016), and the 5-year DFS was 63.0% vs. 68.9% (*p* = 0.019). Kim et al. in their study on 314 rectal cancer patients reported that the 3-year OS and DFS rates in EPC patients were significantly lower than that of no-EPC patients (81.2% vs. 96.2%; *p* = 0.001 and 62.9% vs. 76.1%; *p* = 0.037) [32]. Wan et al. using a cohort of 1513 surgically resected colorectal cancer patients (447 rectum), reported that EPC (≥400 × 10^9^/L) measured within 1 month before surgery was an independent negative prognostic factor of OS (HR = 1.66; 95% CI = 1.34–2.05; *p* = 2.6 × 10^− 6^), and of distant recurrence (HR = 2.81; 95% CI = 1.67–4.74, *p* = 1.1 × 10^− 4^) [35]. Similarly, Sasaki et al. reported, in a study on 636 colorectal cancer patients (222 rectum), that preoperative EPC (> 370 × 10^9^/L) was an independent negative prognostic factor of disease specific survival (HR 3.04; 95% CI 1.82–4.96; *p* < 0.001) [27]. Cravioto-Villanueva et al. reported in a study on 163 rectal cancer patients that preoperative high platelets count associated with poor OS (p < 0.001) [36]. In a study on 629 patients (341 rectum), Nyasavajjala et al. found no difference at multivariate analysis in OS based on preoperative thrombocytosis. In this retrospective study however, the platelets count cut off was set at > 450 × 10^9^/L accounting for a small number of cases with thromboyctosis (8.1%). Moreover, the tumor site (colon vs. rectum) was not imputed as a covariate at multivariate analysis so that no conclusion can be inferred about prognosis in rectal cancer [37].

In our study, EPC was associated with a lower pCR rate, as well as unfavorable long-term oncological outcomes. Some clinical and experimental evidences may help to explain these results. For example, biologically more aggressive tumors have shown the capability of inducing PLT production, which in turn may have an active role in facilitating cancer progression and dissemination by different mechanisms such as protection from immune surveillance, cancer-cell arrest in the micro-vasculature, and neoangiogenesis stimulation [12–15].

The “malicious” role of PLT activity in cancer development might, at least in part, explain the anticancer effect of aspirin use, as proposed in some recent studies. Specific to rectal cancer, a recent prospective non-randomised study looked at the outcome of patients who were taking aspirin during CRT for rectal cancer compared to patients not taking aspirin. Patients in the aspirin arm had a better progression-free survival, mainly driven by a lower incidence of metastasis during follow-up (11% vs. 25%, HR = 0.30, 95% CI = 0.10–0.86). Downstaging of the primary tumour was also increased from 44 to 68% (*p* = 0.011), representing an absolute increase of 24% [38].

From a strict prognostic point of view, it is known that rectal carcinomas not responding to CRT, display a more aggressive clinical behavior, expressed by a higher tendency to develop local and distant recurrence [6, 7]. This data is confirmed by the results of our survival analysis showing a significant worse oncologic outcome in the subgroup of patients with no-pCR. Since increased PLT production and activation appear to represent a cancer cell evolutionary strategy, even in case an active role of PLT in CRT resistance is not confirmed, PLT count might still be used to early identify a subset of LARC patients with less favorable outcome to be proposed for more aggressive alternative therapeutic strategies possibly including anti-platelet approaches [39].

## Conclusions

With the limitation of a retrospective study, our findings indicate that in LARC patients EPC before neoadjuvant CRT is independently associated with lower pCR rate and worse long-term oncologic outcome. This observation is of potential clinical relevance, since it might help in the selection of patients to be proposed for more aggressive therapeutic strategies, as well as for trials using platelet targeting agents.

## Abbreviations

CRT: Chemoradiation therapy
DFS: Disease free survival
EPC: Elevated platelet count
LARC: Locally advanced rectal cancer
OS: Overall survival
pCR: Pathological complete response
PLT: Platelets
TME: Total mesorectal excision
